# Trade-off between resistance and persistence in high cell density cultures

**DOI:** 10.1128/msystems.00323-25

**Published:** 2025-06-13

**Authors:** F. Beulig, J. Bafna-Rührer, P. E. Jensen, S. H. Kim, A. Patel, V. Kandasamy, C. Steffen, K. Decker, D. Zielinski, L. Yang, E. Özdemir, S. Sudarsan, B. Palsson

**Affiliations:** 1 Novo Nordisk Foundation Center for Biosustainability, Technical University of Denmark 587234, Kongens Lyngby, Denmark; 2 Department of Bioengineering, University of California, San Diego, California, USA; Rice University, Houston, Texas, USA

**Keywords:** *Escherichia coli*, high cell density physiology, persistence, resistance, stimulons

## Abstract

Bacterial growth to high cell densities is commonly observed in infections, microbiomes, and biomanufacturing, yet there is limited understanding of how transcriptional regulation changes to balance between growth and survival under associated stresses. Here, we utilized well-controlled culturing systems to grow wild-type and metabolically engineered *Escherichia coli* strains into high cell densities (50–80 g_cell dry weight_ L^−1^) and determine the associated transcriptional dynamics. Knowledge-enriched, machine-learning-based data analytics reveal distinct stress-related gene expression patterns that are consistent with a fundamental trade-off between resistance and persistence. We suggest that this trade-off explains observed growth arrests in high-density cultures and that it results from the disruption of cellular homeostasis, due to re-allocation of limited cellular resources from resistance functions toward increased maintenance requirements. This study deepens our understanding of high-density physiology, illustrates a common link between biomanufacturing and infection, and suggests that persistence phenotypes are programmed as hidden transcriptional states in *E. coli*.

**IMPORTANCE:**

This study presents the first systems-level characterization of *Escherichia coli* high-cell-density physiology, using an integrated experimental and computational approach. Knowledge-enriched, machine-learning-based analysis of the >470 transcriptomic samples reveals distinct stress-related gene expression patterns that allow the first functional and quantitative description of associated stimulons. The identified stimulons reveal a hitherto undiscovered trade-off between resistance- and persistence-like functions. Our findings have fundamental implications for genome editing of strains optimized for resilience toward stresses of cell-dense environments, particularly those encountered in biomanufacturing.

## INTRODUCTION

A comprehensive understanding of high cell-density microbial physiology has remained elusive despite broad and fundamental importance, such as in the context of infectious diseases and industrial biotechnology. For example, achieving high cell densities is key to the economic viability of many biomanufacturing processes that are being developed to support sustainable lifestyles (1, 2). Unfortunately, detailed experimental interrogation of microbial physiology at high cell densities has been hampered by the lack of suitable methodologies but is now enabled by advancements in integrated “omics” approaches and the development of highly controlled, parallelized bioreactors (2).

Dense microbial populations face various sources of stresses not experienced at low densities that, among others, include transient shortage of carbon and nutrients and accumulation of inhibitory or toxic metabolites, such as reactive oxygen species (ROS) (3–7). Stresses need to be balanced with other vital cellular processes to maintain viability. Due to the likelihood of encountering a linked set of adverse challenges, microorganisms are equipped with a global stress response that confers resistance against a variety of stresses, even those not yet encountered or seemingly unrelated (8). Independent of the general stress response, some stress response pathways enable cells to address individual stressors by selectively activating specific gene sets (9, 10). However, maintaining resistance comes at great metabolic cost, and the upregulation of stress response genes typically results in a concurrent downregulation of genes involved in growth-associated processes (10–12). Indeed, “resistant” phenotypes have been shown to be outcompeted by “sensitive” phenotypes that do not incur the maintenance cost of constitutive stress readiness (13). If resistance response functions are exhausted, persistence remains as an alternative survival strategy. Unlike resistance, persistence is a transient physiological state that is characterized by a mixture of growing and growth-arrested cells that result from bistable expression states within a population. Persistence allows cells to adapt and resume growth after an otherwise lethal stress exposure, such as antibiotic treatment (14, 15).

*Escherichia coli*, an extensively studied and widely used workhorse in biotechnology, can achieve >100 g_cell dry weight_ L^−1^ of cell density (3, 4). The natural biosynthetic capabilities of *E. coli* have been expanded by introducing heterologous pathways to develop new biosynthetic pathways. However, engineered pathways may disturb the intracellular processes and resource distribution that have been optimized by natural evolution (16, 17). The associated metabolic burden reduces fitness, as limited cellular resources must be reallocated to maintain physiological and biochemical homeostasis (17, 18). Thus, cellular productivity remains difficult to predict, and growth arrests can be observed, especially at high cell densities.

It remains uncertain how dense bacterial populations balance growth and survival functions. The primary objective of this study was to investigate physiological responses associated with high cell density, especially under metabolic burden, as it is relevant for production strains. We grew different *E. coli* strains in parallel, in well-controlled bioreactor environments to high cell densities—conditions that inherently impose resource limitation—and utilized knowledge-enriched, machine-learning-based analysis to facilitate interpretation of complex transcriptomic responses. Our novel findings reveal that, at high cell densities, the degree of metabolic burden modulates previously unrecognized growth-versus-survival transitions.

## RESULTS AND DISCUSSION

### Maintenance requirements distinguish growth of *E. coli* wild-type (WT) and engineered strains into high cell density

**Fig 1:**
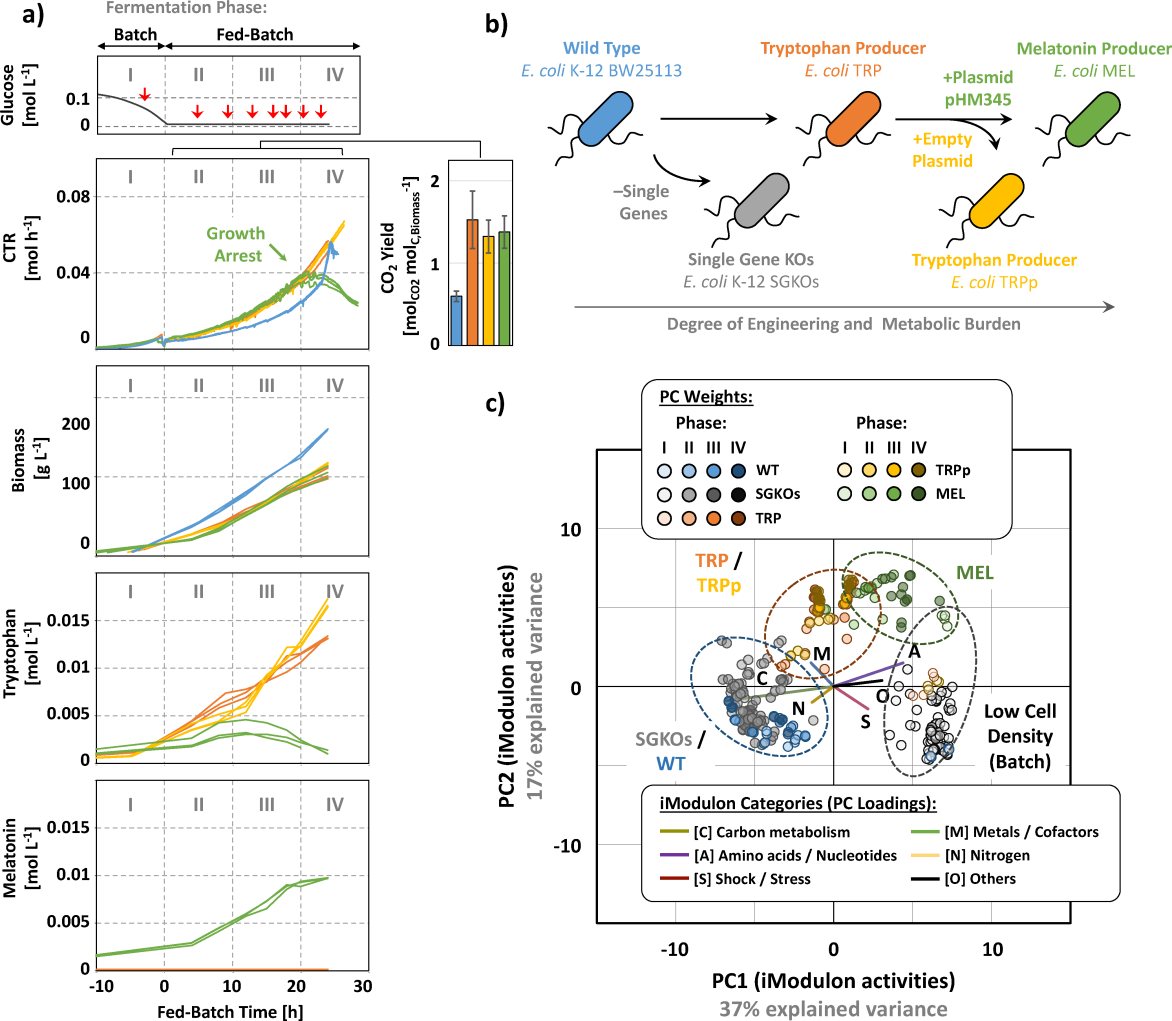
Integrated process and transcriptome diagnostics of high cell-density fermentations. (**a**) Representative time profiles of fermentations from batch (phase I) to fed-batch (phases II, III, and IV) with *E. coli* WT reference strain (blue), as well as genome-engineered production strains TRP (orange), TRPp (yellow), and MEL (green). Red arrows indicate time points for RNA-Seq sampling (see Materials and Methods and https://github.com/febedtu/hd_ecoli for details on RNA-Seq and process data). (**b**) Schematic outline of strain lineage and metabolic burden. (**c**) Biplot of principal components 1 (PC1) and 2 (PC2) from transcriptome samples at different stages of the fermentation.

High cell-density cultivation experiments were conducted following a common two-stage, batch-to-fed-batch, fermentation strategy in parallel sets of well-controlled bioreactors (Fig. 1a). We studied five groups of *E. coli* strains with varying degrees of genome editing (Fig. 1b; see Materials and Methods for details), including a wild-type BW25113 control strain (WT), 31 different single gene knockout strains (SGKO), two tryptophan production strains (TRP and empty plasmid control TRPp), and a plasmid-carrying melatonin production strain (MEL). The production strains were chosen as models for studying host responses to metabolic burden as they represent well-characterized iterations of industrially relevant systems that express complex and metabolically demanding biosynthetic pathways (19). After initiation of the fed-batch phase, growth was maintained with exponential feeding strategies, providing a continuous supply of media, O_2_, and pH buffering capacity to all strains (see Materials and Methods for details). We inferred four major fermentation phases (I–IV; Fig. 1a), corresponding to the transition from batch to fed-batch mode (I/II, 0 h), changes in melatonin and tryptophan formation (II/III, 10 h), and growth arrest of strain MEL (III/IV, 20 h).

Over the time course of cultivation, the increase in cell density was most rapid for the WT strain, with maximum biomass concentrations (average ± standard deviation) of 80 ± 3 g_cell dry weight_ L^−1^ for WT (235 ± 9 OD_600_; *N* = 6) and 51 ± 4 g_cell dry weight_ L^−1^ for production strains (150 ± 12 OD_600_; *N* = 18). In contrast, CO_2_ production of WT strains was about 30% lower than production strains. Melatonin and/or tryptophan production accounted for <1% of the carbon from oxidized glucose in production strains. Thus, as evidence for the metabolic burden imposed by the engineered pathways, all genome-engineered strains sustained maintenance requirements by allocating almost twice as much of the carbon and energy from glucose oxidation toward catabolism (CO_2_ yield per formed biomass; Fig. 1a). Consistent with a growth arrest toward the end of the fed-batch fermentation, CO_2_ formation of MEL plateaued at the end of phase III at 0.04 mol_CO2_ h^−1^, and, following this trend, both melatonin and tryptophan production substantially decreased.

### Contrasting transcriptome structures of *E. coli* wild-type and production strains at low and high cell density

To resolve the dynamics in transcriptome composition (i.e., transcriptome re-allocation) of WT and production strains from low to high cell densities, we obtained a total of 470 transcriptome samples with at least 16 replicated samples over the course of each fermentation (Fig. 1a). For an initial exploration of differences in gene expression patterns among the strains, we reduced the dimensionality of all transcriptomic data sets with principal component analysis (Fig. 1c). The first two principal components, together explaining 54% of expression variability, revealed consistent, condition- and strain-specific gene expression patterns that resulted in clusters representative for low cell-density samples (batch phase) of all strains or high cell-density samples (fed-batch) of individual strains. One of the fed-batch clusters was shared by samples of strains TRP and TRPp, highlighting that both strains share similar global transcriptome allocation. SGKO strains shared a cluster with the WT strain. Overall, the strongest transcriptional changes occurred during the transition from low to high cell density (transition phase I/II), coinciding with the onset of substrate limitation, whereby each strain responded differently to the transition.

Since principal component analysis fails to capture sufficient detail in transcriptome dynamics, we utilized knowledge-enriched independent component analysis to extract iModulons (unique sets of independently modulated genes; see Materials and Methods for more details). By reducing the high-dimensional nature of transcriptome data into a smaller set of biologically meaningful gene expression patterns that reflect the transcriptional regulatory network, iModulons facilitate interpretation of complex transcriptomic responses (12). Among all analyzed strains and fermentation phases, iModulons captured >90% of genes that were significantly upregulated (179 ± 34; Δlog tpm > 2, false discovery rate [FDR] < 0.1) or downregulated (77 ± 22; Δlog tpm <–2, FDR < 0.1). Out of 194 iModulons, 48 were considered putatively high-density specific with >5 difference in activity low cell-density samples (|ΔA| > 5, FDR < 0.1). Between the different media compositions, “High Fe/Zn” and “Low Fe/Zn” only zinc and motility-associated FliA/FlhDC iModulons were highly active (|ΔA| > 5, FDR < 0.1), suggesting an essential role of zinc in the expression of genes involved in flagellar synthesis. Consistent with principal component analysis-based clustering (Fig. 1c), a comparison of TRP against TRPp samples did not reveal differences in activated iModulons (|ΔA| > 5, FDR < 0.1). A comparison of SGKO against WT samples revealed an activation of sequence insertion elements (IS1 iModulon). As will be discussed in the following sections, several of the putative high-density-specific iModulons showed systematic variability over the course of the fermentation, revealing underlying regulatory mechanisms. We could not identify a pronounced correlation of iModulons with OD (|Spearman *R*^2^| > 0.7, *P* < 0.05), possibly due to the absence of universal, continuous relationships of iModulon activities with cell density itself. Differential iModulon activity during growth phases I–IV thus reflected transcriptome re-allocation with increasing OD and the shift of unrestricted growth to a regime of controlled growth limited by substrate supply (transition phase I/II).

**Fig 2:**
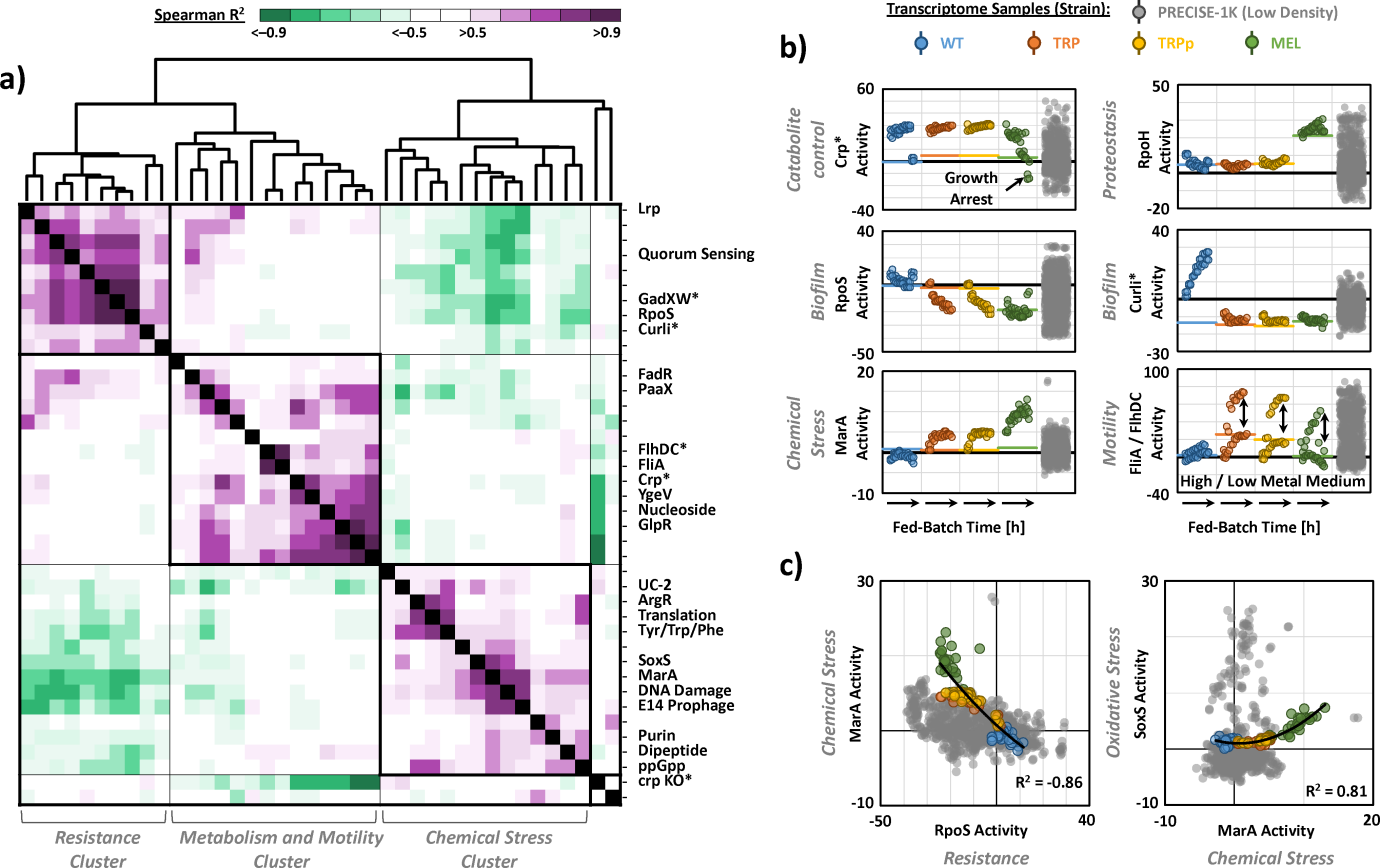
iModulon analysis reveals a trade-off between resistance and persistence functions. (**a**) Spearman correlation matrix for activity levels of 48 high cell-density-specific iModulons with significantly higher or lower activity in fed-batch phase as compared to the batch phase (|ΔA| > 5, FDR < 0.1; see Materials and Methods and https://github.com/febedtu/hd_ecoli for details, such as iModulon composition). Clusters representing stimulons were named according to iModulons with largest size (number of genes) and most significant correlations (>10 connections are highlighted). (**b**) Activity of major iModulons over fed-batch time. Horizontal lines represent average activity during the batch phase. Vertical arrows indicate FliA/FlhDC activity difference between cultivations with low and high metal supplementation, suggesting their role as essential motility cofactor. iModulons denoted with an asterisk represent the sum of iModulons with the same annotation but differing suffix (i.e., Crp*, crp KO*, Curli*, FlhDC*, and GadXW* represent the sum of Crp-1/2, crp KO-1/2, Curli-1/2/3, FlhDC-1/2, and GadXW-1/2, respectively). (**c**) Representative iModulon correlations for global resistance, chemical stress, and oxidative stress.

To reveal functional associations and underlying regulatory relationships that govern stress-related responses at high cell densities, we searched the transcriptome data sets of all investigated strains for highly significant correlations among the 48 activated iModulons (|Spearman *R*^2^| > 0.7, *P* < 0.05; Fig. 2a). We detected three major stimulon clusters, represented by (i) RpoS and GadXW (resistance-related), (ii) Crp and FlhDC (metabolism- and motility-related), and (iii) MarA and SoxS (chemical stress-related) as their largest and most connected iModulons, respectively. The broad variability of detected iModulon activity changes allowed us to robustly separate the transcriptomic state of individual strains, as it captured a substantial portion of gene expression states in the background data set (>1,000 RNAseq profiles; PRECISE-1K) (20). Furthermore, the deviation of iModulon–iModulon activity correlations from the background data set, generated from low-density cultures, highlights both strain- and high cell-density-specific gene regulatory dynamics. In the following sections, we will provide a systems-level description of how coordinated iModulon activities represent well-defined molecular processes that reflect a novel trade-off between resistance- and persistence-like stress responses governing high cell density physiology.

### Knowledge-enriched transcriptome analysis reveals trade-off between resistance and persistence

In *E. coli*, the general stress resistance mediator is σ^38^, RpoS, a sigma subunit of RNA polymerase that partially replaces housekeeping sigma factor σ^70^, RpoD, to regulate more than 500 genes that allow rapid responses to threatening environmental conditions (8, 9, 21). We identified an upregulation of the RpoS iModulon in the WT strain and distinct positive correlations with multiple stress- and resistance-related iModulons, including GadXW, Suf System, RpoE, NRZ, and Quorum Sensing (Spearman *R*^2^ >0.7), that are consistent with a general stress readiness (resistance stimulon; Fig. 2a). For example, acid stress is one of the most common environmental stresses for *E. coli*, and, although the cultivation was performed under controlled pH conditions, the WT strain activated GadXW iModulons containing central components of the glutamate decarboxylase acid tolerance system (8). The WT strain also coordinated upregulation of antioxidant systems such as catalases KatE, KatG, and DNA-binding ferritin Dps, as part of the RpoS iModulon, with reactive Fe-S cluster assembly as part of the Suf System iModulon (Spearman *R*^2^ = 0.87) (22, 23).

**Fig 3:**
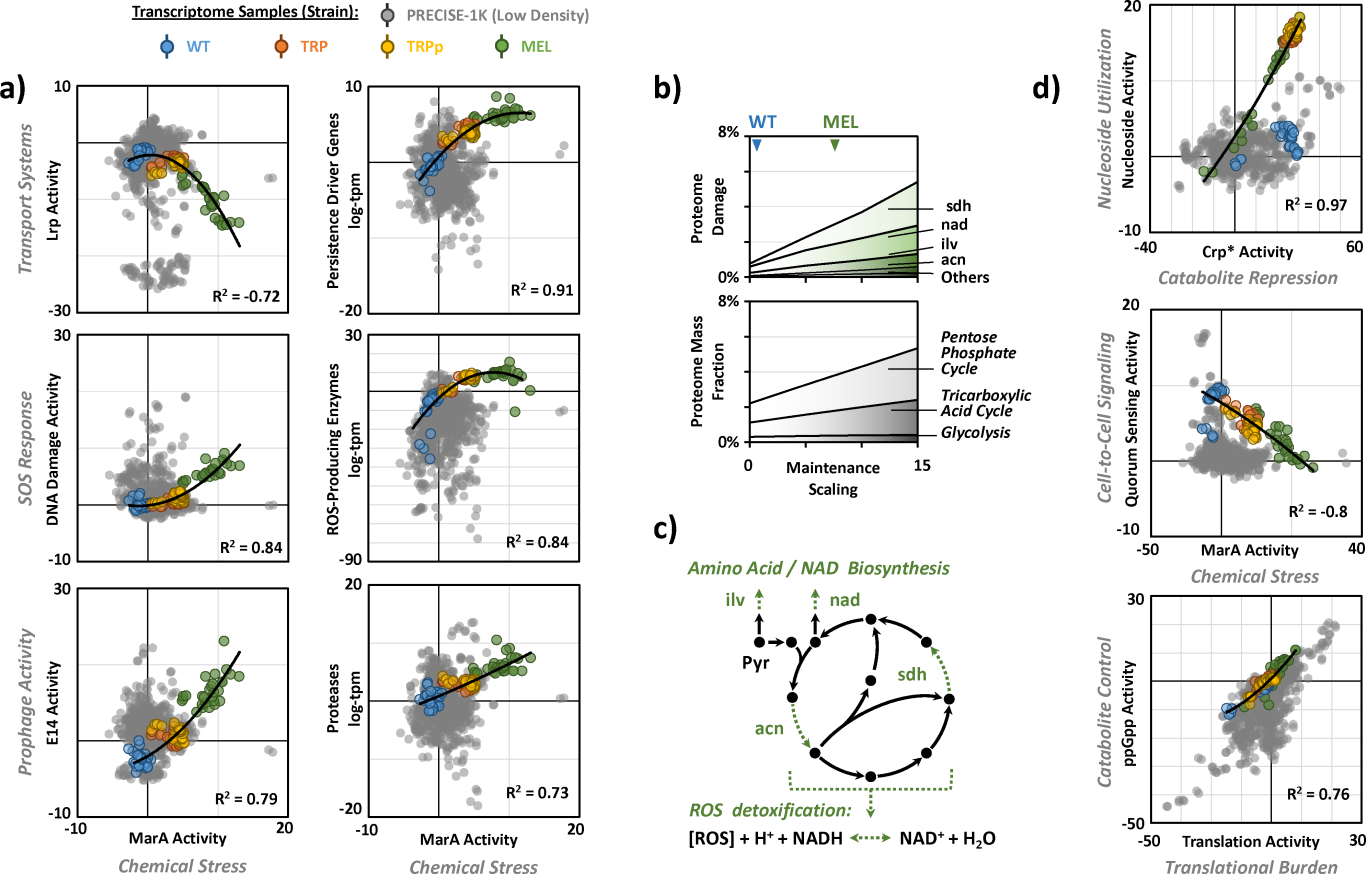
Chemical stress modulates persistence at high cell density. (**a**) Representative iModulon and gene set correlations for chemical stress and persistence functions. Genes encoding ROS-producing enzymes, persistence drivers, and proteases are listed in Table S1. (**b**) Proteome allocation for central carbon metabolism and proteome damaging reactions, as calculated with the genome-scale OxidizeME model. (**c**) Predicted damage to enzymes associated with the tricarboxylic acid cycle is highlighted in green. (**d**) Representative iModulon correlations for cellular communication and growth functions.

RpoS and associated resistance-related iModulons were significantly downregulated in production strains (Fig. 2b and c), and, as part of a distinct negative correlation (Spearman *R*^2^ = −0.86), we identified the concurrent upregulation of chemical stress-related iModulons MarA and SoxS (chemical stress stimulon; Fig. 2a and c). In fact, MarA activities in MEL exceeded those in almost the entire low-density background data set. Together with SoxS, MarA acts as a transcriptional regulator of overlapping regulons that contain genes involved in stress response to inhibitory and toxic chemical species, including antibiotics and redox cycling compounds, such as ROS (24). As evidence of the cells’ attempt to shield themselves from harmful chemicals, we found an upregulation of multidrug exporters MdlAB and AcrAB, as part of the SoxS iModulon, concurrent to a downregulation of porins and diverse uptake systems in the Lrp iModulon that is mediated by non-coding RNA *micF* under regulatory control of SoxS (Fig. 3a) (9, 24).

In the absence of resistance or tolerance functions, severe stress signals may trigger persistence and associated growth arrest as an alternative survival strategy (14, 15, 25, 26). At its core, persistence is a phenotypic response to strong perturbation of metabolic homeostasis, involving multifactorial molecular mechanisms (14, 27). The most significant persistence triggering mechanisms involve sensitization through suppression of global resistance (RpoS response), as well as stress responses toward starvation (nutrient and ATP limitation), disruption of proteostasis (heat shock response), DNA damage (SOS response), and inhibitory or toxic chemicals (multidrug response) (14, 15, 25–33). Indeed, production strains upregulated recently identified persistence “drivers” (genes contributing to the formation or maintenance of persistence) and ”markers” (genes with distinct expression during persistence) with distinct positive correlation to chemical stress-related iModulon MarA (see Materials and Methods for details; Spearman *R*^2^ >0.9; Fig. 3a) (34). So far, persistence has not been reported to be of relevance in bioprocess systems. Yet, we found recurring evidence for activation of persistence-mediating mechanisms in the investigated production strains at high cell densities, as described in the following sections.

### Carbon and energy limitation promote chemical stress at high cell density

The transition from low to high cell densities is inevitably accompanied by a transition from unrestricted growth to growth under substrate limitation and a necessity to rebalance catabolism and anabolism to support heightened maintenance requirements and biosynthetic needs (35, 36). *E. coli* responds to carbon and energy limitation by synthesizing cAMP and the associated receptor protein Crp (35). Thus, at the start of the fed-batch phase, all strains upregulated multiple cAMP-Crp-regulated iModulons (metabolism and motility stimulon cluster; Fig. 2a), containing more than 200 genes that allow catabolic flexibility under carbon starvation. Consistent with the detected growth arrest of MEL, the activity of all cAMP-Crp-regulated iModulons diminished toward the end of the fermentation (Fig. 1a and 2b).

High-density cultures must produce a significant amount of energy to meet their metabolic demands, which result in continuous formation of toxic radicals, as about 0.5% of consumed oxygen inevitably turns into ROS that cause damage to vital macromolecules, such as DNA, RNA, proteins, and lipids (22, 37). Indeed, we found the highest expression of ROS-producing enzymes in the production strains, positively correlated with chemical stress-related iModulon MarA (Spearman *R*^2^ = 0.84; Fig. 3a). To characterize the impact of ROS on WT and production strain metabolism *in silico*, we utilized OxidizeME, a genome-scale computational model of *E. coli* metabolism and expression, which incorporates a multiscale description of ROS stress response and proteome damage (38). Consistent with higher catabolic fluxes in production strains (Fig. 1a), OxidizeME predicts a sixfold increase in maintenance cost over the WT strain, and the associated demand for ATP and reducing equivalents is supported by an enhanced flux into the pentose phosphate cycle and tricarboxylic acid cycle (Fig. 3b and c). However, higher catabolic fluxes also lead to a proportional increase in ROS generation and associated damage to the proteome. If the calculated intracellular production of ROS (~90 µmol L_cell_^−1^ s^−1^) outstrips the cell’s NADH-dependent capacity for detoxification and ATP-dependent repair capacity, e.g. due to transient substrate limitation at high cell densities, intracellular ROS accumulation would quickly breach toxic thresholds and lead to growth arrest (>0.5 µmol L_cell_^−1^) (22).

### Genotoxic and proteotoxic stress modulate persistence at high cell density

Unmitigated exposure to oxidative stress appeared to trigger SOS responses resulting in continuously increasing activity of the DNA Damage iModulon over the course of the fermentation, especially in production strain MEL (Fig. 3a). The SOS response modulates bacterial persistence by acting as a signaling pathway (regulated by LexA) that complements other stress responses and by providing DNA repair functions that are essential for transitioning back from dormancy to a metabolically active state (33). MEL not only activated “weaker” SOS functions (low LexA repressor affinity), such as Uvr-dependent DNA repair, but also “stronger” SOS functions (high LexA repressor affinity), such as Umu-dependent mutagenesis repair and SulA-dependent growth arrest (39, 40). Furthermore, we found activation of the E14 iModulon, containing cryptic lambdoid prophage elements (Fig. 3a). Under stress, cryptic prophages have been shown to provide diverse benefits for survival, and E14 is among the most commonly activated, especially during the SOS response (41). Although prophages do not appear to influence persister formation, they inhibit their transition back to a metabolically active state (42).

Perturbation of protein homeostasis (e.g., by chemical stress) favors the formation of protein aggregates, which, apart from their toxicity, facilitates persister cell formation (28, 34, 43). Indeed, the expression of proteases, as modulators of translational deficiencies, strongly correlates with the expression of persistence marker and driver genes (Spearman *R*^2^ > 0.83), as well as MarA activity (Spearman *R*^2^ = 0.73; Fig. 3a). Consistent with challenges to maintain proteostasis during heterologous protein expression, we found high activity of the associated RpoH iModulon for MEL compared to WT during all phases of the fermentation (|ΔA| > 5, FDR < 0.1). RpoH controls multiple translational control systems, of which persistence markers *ibpA* and *ibpB* were among the highest expressed genes in the entire transcriptome data set (11 ± 0.5 and 11.5 ± 0.3 log tpm, respectively). These chaperones are reported to be exclusively upregulated during extensive protein aggregation, to attenuate the cell’s global stress response, and to impede protein disaggregation (28, 34, 44). Upregulation of RpoH is often accompanied by downregulation of RpoS, allowing for the redirecting of cellular resources to support the energy-intensive functionality of ATP-dependent chaperones and proteases (45, 46).

### Impact of high cell density on cell-cell communication, motility, and biofilm formation

During substrate limitation, *E. coli* adopts ATP-mediated intercellular communication that results in pronounced heterogeneity of intracellular ATP concentrations, whereby accumulation of ATP in a small fraction of the population is supported by starvation of the remaining cells (47, 48). In our study, production strains upregulated genes that are implicated in the utilization of extracellular ATP as part of the Nucleoside iModulon, which, indicative of substrate starvation signals, correlated with cAMP-Crp-related iModulons (Spearman *R*^2^ = 0.97; Fig. 3d). As highlighted earlier, starvation is an important trigger for persistence (30–32). The nucleoside iModulon includes the channel-forming protein and permeases (*tsx* and *nup* operon), as well as associated salvage pathway proteins (*cdd*, *udp*, and *deo* operon). As the transmission of ATP is localized, ATP-based intercellular communication is expected to become only relevant at growing cell densities. Quorum sensing, as another form of intercellular communication, was primarily expressed in WT samples at high cell densities (Fig. 3d). However, we did not observe a significant correlation of quorum sensing to cell density itself but a distinct negative correlation with MarA (Spearman *R*^2^ = −0.8) that is likely mediated through repression of biofilm formation, distributed across the Curli iModulons (7, 24).

The inverse relationship between motility and biofilm formation is regulated by competition of RpoS with flagellar sigma factor σ^28^, FliA, and dependence of biofilm-forming curli synthesis on the downregulation of the flagellar regulatory cascade (fight-or-flight strategy) (7). Consequently, an upregulation of flagellar assembly and chemotaxis, as part of the FlhDC and FliA iModulons, was restricted to production strains (Fig. 2b). While flagellar motility can be associated with persistence functions, it remains unclear whether its upregulation in the production strains is a driver or simply a marker of stress responses. One link lies in the energy expenditure of flagellar motility, which leads to increased production of ROS (37). Another link lies in cell density- and communication-mediated chemical stress signaling (49). AcrA, upregulated in production strains as part of the SoxS iModulon (Fig. 2c), was previously identified as a “necrosignal” that is released from motile, antibiotic-killed cells to communicate an emergency state and induce persistence functions in the surviving population (49, 50).

### Molecular mechanisms modulating resource allocation at high cell densities

**Fig 4:**
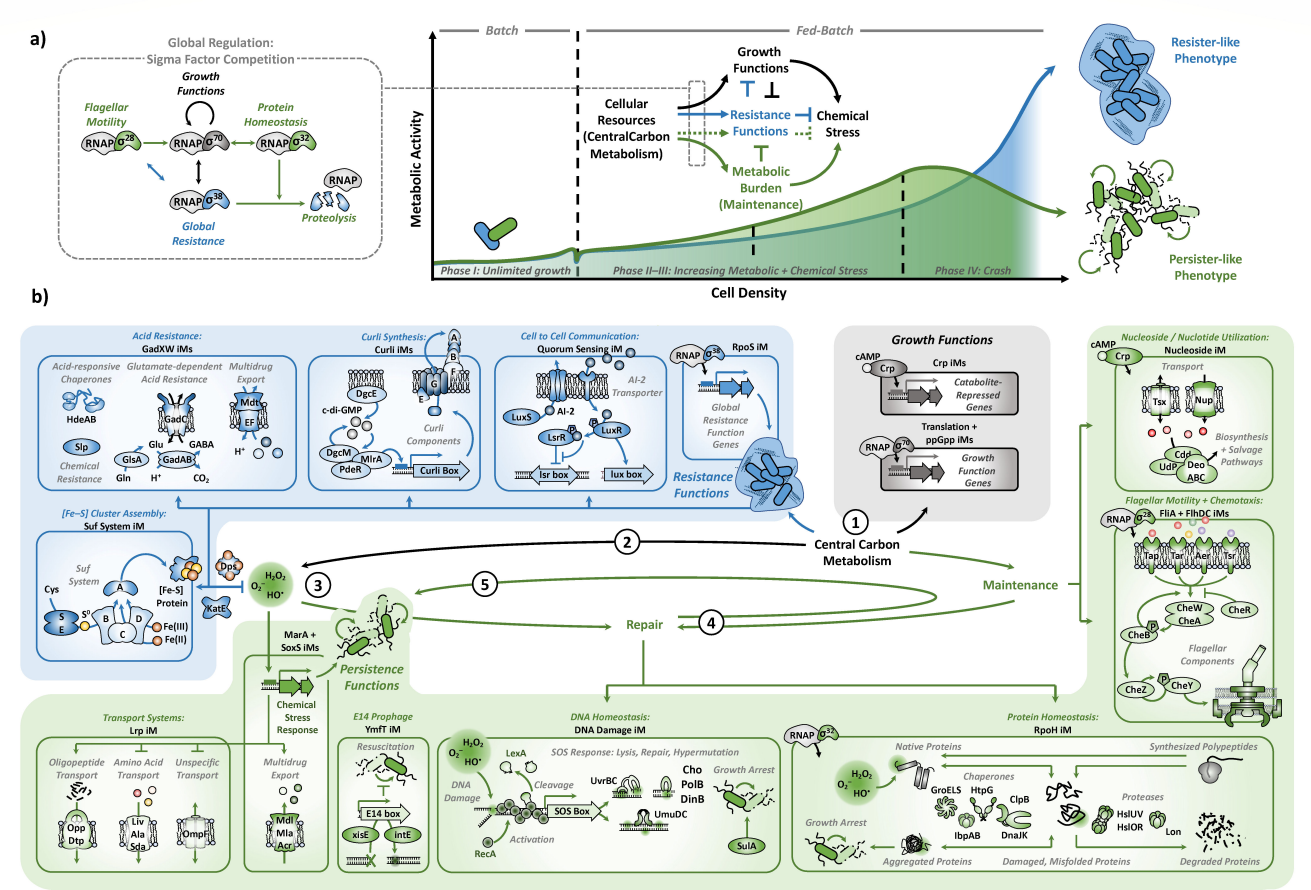
Interaction of cellular processes that are independently regulated during growth to high cell densities (resource-limited fed-batch conditions). Graphical summary of (**a**) the sigma factor regulatory network controlling distribution of cellular resources and (**b**) molecular processes, represented by activated iModulons, shaping the resistance-versus-persistence trade-off at high cell densities: (1) limited cellular resources are distributed between growth, resistance, and maintenance; (2) demand for cellular resources is supported by high flux through central carbon metabolism, leading to production of ROS; (3) oxidative stress is either buffered (resister-like phenotypes) or activates chemical stress functions (persister-like phenotype); (4) accumulated damage to macromolecules necessitates resource-intensive repair functions, increasing maintenance requirements; (5) exceeded repair capacity triggers growth arrest. Black arrows represent regular growth functions in all strains. Blue arrows and boxes represent processes primarily linked to resistance functions (strain WT). Green arrows and boxes represent processes primarily linked to persistence functions (strain MEL).

Due to limited cellular resources, microorganisms cannot maximize proliferation and stress resistance at the same time (11). This fundamental constraint is expressed in the trade-off between the RpoS iModulon and RpoD-regulated iModulons (“fear vs greed”) that, on a molecular level, follows the competition between available sigma factor subunits for a limited number of RNA polymerase molecules (Fig. 4a). Specifically, the two growth-related iModulons ppGpp and Translation, together comprising 132 genes that are mostly downregulated by ppGpp and/or upregulated by RpoD, govern essential processes associated with protein synthesis and ribosomal function. Their positive correlation (Spearman *R*^2^ >0.76; Fig. 3d) represents the transcriptional blueprint for the allocation of limited cellular resources toward proliferation (growth or maintenance functions), highlighting a more “offensive” strategy in production strains, as opposed to a “defensive” withdrawal of precursors toward resistance functions in the WT strain (Fig. 4a and b). Thus, production strains appear to prioritize outgrowing any damage accumulating resulting from a lack of resistance functions, while navigating the constraints imposed by the necessity for resource-intensive maintenance and repair functions.

### Conclusions

Integration of knowledge-enriched, machine-learning-based analysis allowed us to systematically dissect the fundamental transcriptomic structure of a novel trade-off between resistance- and persistence-like functions. This trade-off is modulated by stimulons, i.e., the coordinated activation of associated iModulons, each representing well-defined molecular processes (Fig. 4a and b). Our findings reveal that, at high cell densities, *E. coli* employs multiple layers of regulation that decide between resource-intensive investment in stress protection or maximizing growth at the risk of breaching proteotoxic and genotoxic thresholds. Oxidative stress and substrate limitation render cells susceptible to persister cell formation and growth arrest. Thus, positioning on the resistance-to-persistence trade-off appears to be, at its core, dependent on resource allocation toward the weight of metabolic burden (WT < TRP ≈ TRPp < MEL). As an emergent property in dense microbial populations, persistence has been extensively studied in the context of infectious diseases and antibiotic resistance (15, 26, 49, 50). However, its relevance in biomanufacturing environments has so far remained unrecognized. Consequently, our findings establish persistence and associated triggers and/or regulators as key targets for the design of strains that show resilience toward stresses of resource-limited, cell-dense fermentation environments. This study provides the first system-level understanding of high cell-density *E. coli* physiology and reveals complex links between transcriptome dynamics and metabolic engineering that are distinct from those at low cell densities.

## MATERIALS AND METHODS

### Bacterial strains and cultivation setup

Batch-to-fed-batch fermentation experiments were performed in 0.25 L Ambr fermenters (Sartorius) with two different media compositions (“High Metal” and “Low Metal” medium) and four strains, including strain WT (*E. coli* strain BW25113) and *E. coli* BW25113-derived production strains TRP (HMP3071, tryptophan producer), TRPp (SDT551, tryptophan producer), and MEL (HMP3427, melatonin producer) with genotype *folE*(T198I), *ynbB*(V197A), Δ*tnaA*, Δ*trpR*(P2–ckDDC), *obgE*(E350A), Δ*yddG*::PJ23101–sgAANAT(D63G), Δ*pfolE*::PJ23100, Δ*ldhA*::PJ23101–sgAANAT(D63G), *trpE*(S40F), and Δ*fhuA*, allowing S-adenosyl methionine cycle-dependent production of tryptophan (19). Strain MEL also carried production plasmid pHM345 (genotype J23107:*tph–pcd–asmt* Kan^R^), allowing further conversion of tryptophan to melatonin, whereas strain TRPp carried empty control plasmid pSD124 (genotype Kan^R^) (19).

The “Low Metal” batch media contained base medium (22 g L^−1^ D-(+)-glucose monohydrate, 7.6 g L^−1^ (NH_4_)_2_SO_4_, 15.7 g L^−1^ KH_2_PO_4_, 0.241 g L^−1^ MgSO_4_, 100 mg L^−1^ thiamine hydrochloride, 0.5 mL L^−1^ Antifoam 204), and 1 mL L^−1^ of trace metal solution (0.48 g L^−1^ CuSO_4_·5H_2_O, 0.54 mg L^−1^ CoCl·7H_2_O, 0.54 g L^−1^ ZnSO_4_·7H_2_O, 2 g L^−1^ CaCl_2_·2H_2_O, 41.8 g L^−1^ FeCl_3_·6H_2_O, 0.3 g L^−1^ MnSO_4_·H_2_O, 33.4 g L^−1^ Na_2_EDTA, 0.5 g L^−1^ Na_2_MoO_4_·2H_2_O). The “High Metal” batch media contained the same base medium, 3.5 mL L^−1^ trace metal solution, and was additionally supplemented with 1.86 g L^−1^ citric acid monohydrate and 41.8 mg L^−1^ FeCl_3_·6H_2_O, 21.6 mg L^−1^ ZnSO_4_·5H_2_O). Initial batch fermentations were performed with base medium until complete glucose consumption, marked by a 50% decline in the rate of CO_2_ formation, triggering an automatic switch to fed-batch operation with continuous supply of the feed medium (550 g L^−1^ D-(+)-glucose monohydrate, 13.7 g L^−1^ MgSO_4_·7H_2_O), according to the following:


$$F(t)=F_{0}\times e^{\mu_{set}\times t}$$

where *F*(*t*) is the continuous feed rate; *F*_0_ is the initial feed rate (0.535 mL h^−1^); µ_set_ is the set specific growth rate (0.11 h^−1^); and *t* is the process time (4). Ambr fermenters perform online measurement of dissolved O_2_ and exhaust gas O_2_, CO_2_, as well as operating parameters. In our experiments, temperature was set at 30°C, dissolved O_2_ was controlled at 40% (cascade control with stirring speed from 1,000 to 4,000 revolutions per minute, air flow from 0.1 to 0.2 L min^−1^, and O_2_ flow from 0.0 to 0.05 L min^−1^), and pH was maintained at 6.5 with ammonium hydroxide (4 mol L^−1^). Throughout the cultivation, samples were taken periodically by an automated liquid handler for transcriptome analysis, as well as offline analysis of biomass, tryptophan, melatonin, glucose, and organic acid concentrations.

To extend the high-density transcriptome data basis for independent component analysis (described in the following), supplemental batch-to-fed-batch fermentation experiments were performed with single gene knockouts of *E. coli* strain BW25113 (Δ*phoB,* Δ*qseF,* Δ*basS,* Δ*kdpE,* Δ*qseB,* Δ*qseC,* Δ*lsrR,* Δ*phoB,* Δ*qseB,* Δ*baeR,* Δ*lsrB,* Δ*rpoS,* Δ*arcA,* Δ*baeR,* Δ*lsrK,* Δ*luxS,* Δ*tnaA,* Δ*cra,* Δ*csgD,* Δ*fnr,* Δ*fur,* Δ*nac,* Δ*proV,* Δ*prpR,* Δ*purR,* Δ*yieP,* Δ*csgD,* Δ*fur,* Δ*nac,* Δ*prpR,* or Δ*purR*) in 0.25 L Ambr reactors and “Low Metal” medium, following the operational procedures described above.

### Analytical procedures

For the determination of biomass concentration during the fermentation, the optical density at a wavelength of 600 nm of the culture (OD_600_) was measured using a spectrophotometer (Jenway 7205 UV/visible; Cole-Parmer, UK). Measured OD_600_ values were converted to cell dry weight with a correlation factor of 0.34 g L^−1^ OD_600_^−1^. For the measurement of extracellular melatonin and tryptophan analysis, samples were filtered (MultiScreen filter plates with 0.45 µm Durapore membranes; Merck Millipore, Germany) and measured using high performance liquid chromatography (HPLC; Dionex Ultimate 3000, Thermo Fisher Scientific, USA) equipped with a C18 column (Zorbax Eclipse Plus C18, 4.6 × 100 mm, with a pre-column filter; Agilent, USA), and Diode Array Detector (DAD 3000; Thermo Fisher, USA). Separation was achieved using a gradient flow of 1 mL min^−1^ with solvent A (0.02% [vol/vol] acetic acid, 99.9% HPLC grade in ultrapure water) and solvent B (acetonitrile, ≥99.9% HPLC grade). The following elution profile was employed: elution started with 5% solvent A, followed by three steep gradients: first to 12% from 0 to 1.5 min, and elution was maintained until 2.5 min; second to 30% from 2.5 to 4.5 min, and elution was maintained until 5.5 min; third to 70% from 5.5 to 8 min. To equilibrate the column back to its initial conditions, the gradient was decreased to 5% solvent A from 9 to 9.5 min and maintained until the end of run time (11 min). To quantify melatonin and tryptophan, 1 µL of the sample was injected, and the column temperature was kept constant at 30°C. For the analysis of glucose and short-chain fatty acids (acetate, citrate, formate, lactate, and pyruvate), samples were centrifuged for 5 min at 16,200 × *g* at 4°C. The resulting supernatant was diluted 1:10 in 9 mM sulfuric acid solution, filtered (MultiScreen filter plates with 0.45 µm Durapore membranes; Merck Millipore, Germany), and measured using HPLC (Dionex Ultimate 3000, Thermo Fisher Scientific, USA) equipped with an ion exclusion column (Aminex HPX-87X, 300 × 7.8 mm; BioRad, USA), as well as refractive index and UV detectors (RI-150; Thermo Fisher, USA). Separation was achieved using a 9 mM sulfuric acid solution as the mobile phase with an isocratic flow of 0.7 mL min^−1^. To quantify the organic acids, 10 µL of the sample was injected, and the column temperature was kept constant at 60°C.

### RNA sequencing and iModulon analysis

We obtained a total of 470 transcriptome samples. Details on the individual experiments are summarized in the metadata of the data repository at https://github.com/febedtu/hd_ecoli. Briefly, 128 samples were derived from a single highly time-resolved batch-to-fed-batch experiment (4 strains × 8 time points × 2 media × 2 replicates). The remaining 342 samples were obtained from experiments using WT, SGKO, and production strains at different time points under batch and fed-batch conditions (see “Bacterial strains and cultivation setup”). Transcriptome samples were collected and prepared in biological duplicates, whereby either 2 mL of culture (low cell-density culture, batch phase) or 0.5 mL of culture (high cell-density culture, fed batch phase) was added to 6 mL of Qiagen RNA-protect Bacteria Reagent immediately after sample collection. This solution was then vortexed for 30 seconds, incubated at room temperature for 5 min, and then centrifuged. The supernatant was then removed, and the cell pellet was stored at −80°C. The RNeasy Mini Kit (Qiagen) was used to extract and purify RNA from the cell pellets per vendor protocol, including on-column RNase-Free DNase treatment (Qiagen) for 30 min at room temperature. RNA was quantified using a Qubit fluorometer (Thermo Fisher), and integrity was assessed using a fragment analyzer (Agilent Technologies). Ribosomal RNA was removed using the QIAseq FastSelect –5S/16S/23S kit (Qiagen). Sequencing libraries were prepared with the KAPA Stranded RNA-Seq Library Preparation Kit (Kapa Biosystems) and assessed using a fragment analyzer (Agilent Technologies). The libraries were then subjected to paired-end sequencing on Illumina Nextseq using Nextseq Mid Output kit (Illumina) with a read length of 150 bp. The raw sequencing reads were demultiplexed based on sample indexes using BaseSpace (Illumina), and the fastQ files were used for further processing, as described previously (https://github.com/avsastry/modulome-workflow) (12). The resulting, high-quality RNA-sequencing data sets from this study were combined with available, low-density PRECISE-1K data sets (https://github.com/SBRG/precise1k) to compute iModulons by performing independent component analysis, as described previously (12, 20). Briefly, 100 iterations of the OptICA algorithm were performed across a range of dimensionality to find robust components which appeared in more than 50 of the iterations of an individual dimensionality. An independent component dimensionality of 400 was chosen for iModulon reconstruction, annotation, and analysis, following established protocols (https://github.com/SBRG/pymodulon) (12, 20). At the optimal dimensionality, the total number of non-single gene iModulons was 194. Differential iModulon activities were calculated, as described previously (12). For each iModulon, a null distribution was generated by calculating the absolute difference between each pair of biological replicates and fitting a log-normal distribution to them. For the groups being compared, their mean difference for each iModulon was compared to that iModulon’s null distribution to obtain a *P*-value. The set of *P*-values for all iModulons was then FDR corrected to generate q-values. For comparison between samples, a difference in iModulon activity, ΔA, >5 was considered as highly active (FDR < 0.1). RNA-Seq- and iModulon-related data, such as iModulon activity and composition, are available in the data repository at https://github.com/febedtu/hd_ecoli.

### Genome-scale model of metabolism and macromolecular expression (ME model)

We used OxidizeME, a genome-scale model of metabolism and expression with ROS damage responses (38). Models were constrained using phenotypic data (growth rate, glucose uptake rate, oxygen uptake rate, CO_2_ production rate, organic acid production rates) and expression data, as described previously (51). Growth was simulated over a range of maintenance costs (reaction “ATPM”) with basal intracellular ROS concentrations for the WT strain (0.2 nmol L^−1^ superoxide and 50 nmol L^−1^ hydrogen peroxide). As the steady-state level of ROS is proportionate to the rate of its formation, intracellular ROS concentrations of the production strain were scaled according to the increase in oxygen uptake rate relative to the WT strain (37, 38). The percentage of the proteome allocated to glycolysis, pentose phosphate cycle, and tricarboxylic acid cycle cycle was calculated using the solutions from each model, according to the following:


$$\%\text{ proteome }=\left(\sum_{i}{mw}_{i}\times V_{i,\text{translation }}\right)/\left(\sum_{j}{mw}_{j}\times V_{j,\text{translation }}\right)$$

where mw_*j*_ and *V*_*j*,translation_ represent the molecular weight and translation flux of the *i*-th protein in a given pathway, respectively, and mw_*j*_ and *V*_*j*,translation_ represent the molecular weight and translation flux of the *j*-th protein in the entire model. Similarly, the damaged portion of the proteome was calculated, according to:


$$\%\text{ damaged proteome }=\left(\sum_{k}{mw}_{k}\times V_{k,\text{ComplexFormation}}\right)/\left(\sum_{j}{mw}_{j}\times V_{j,\text{translation}}\right)$$

where mw_*k*_ and *V*_*k*,ComplexFormation_ represent the molecular weight and translation flux of all OxidizeME protein damaging reactions (denoted with the prefix “damage_”), respectively.

## Data Availability

RNA-seq data have been deposited to GEO and are publicly available as of the date of publication, under accession numbers GSE252784. Original data and code for analysis are available at https://github.com/febedtu/hd_ecoli and any additional information or code required to reanalyze the data reported in this paper is available from the lead author or correspondence contact upon request.

## SUPPLEMENTAL MATERIAL

Table S1 msystems.00323-25-s0001.docx Functional annotation of key genes associated with ROS production and persistence in *E. coli*.

ASM does not own the copyrights to Supplemental Material that may be linked to, or accessed through, an article. The authors have granted ASM a non-exclusive, world-wide license to publish the Supplemental Material files. Please contact the corresponding author directly for reuse.
