# Supplementary material for: Trade-off between resistance and persistence in high cell density cultures
Source: mSystems. 2025 Jun 13;10(7):e00323-25. doi: 10.1128/msystems.00323-25 (PMC12282090; doi:10.1128/msystems.00323-25)
Supplement: Table S1 — Functional annotation of key genes associated with ROS production and persistence in E. coli. [file msystems.00323-25-s0001.docx]

**Supplementary Table 1: Functional annotation of key genes associated with ROS production and persistence in *E. coli*.**

| Gene | Functional Annotation | COG category* |
| --- | --- | --- |
| *Putative ROS-producing enzyme-coding genes (23, 38)* | | |
| AceE | Pyruvate dehydrogenase | F |
| AsnA | Asparagine synthetase A | F |
| AtpC | ATP synthase F1 complex | C |
| Cmk | Cytidylate kinase | F |
| CycA | Amin Acid permease | E |
| CyoD | Cytochrome bo3 ubiquinol oxidase | C |
| FabH | Beta-ketoacyl-[acyl carrier protein] synthase | I |
| GcvP | Glycine decarboxylase | E |
| GdhA | Glutamate dehydrogenase | E |
| GlyA | Serine hydroxymethyltransferase | E |
| Gnd | 6-phosphogluconate dehydrogenase | F |
| GuaB | Inosine 5'-monophosphate dehydrogenase | F |
| HemF | Coproporphyrinogen III oxidase | H |
| LldP | Lactate/glycolate permease | C |
| Lpd | Lipoamide dehydrogenase | F |
| Mdh | Malate dehydrogenase | C |
| NuoN | NADH:quinone oxidoreductase | C |
| Pgi | Glucose-6-phosphate isomerase | F |
| Pgl | 6-phosphogluconolactonase | F |
| Ppk | Polyphosphate kinase | F |
| ProA | Glutamate-5-semialdehyde dehydrogenase | E |
| ProB | Glutamate 5-kinase | F |
| Pta | Phosphate acetyltransferase | C |
| PtsH | Phosphocarrier protein | G |
| PurN | Phosphoribosylglycinamide formyltransferase | F |
| PyrH | UMP kinase | F |
| SdhC | Succinate:quinone oxidoreductase | C |
| SerA | Phosphoglycerate dehydrogenase | E |
| SerB | Phosphoserine phosphatase | E |
| SucA | 2-oxoglutarate dehydrogenase | F |
| SucC | Succinyl-CoA synthetase | F |
| TpiA | Triose-phosphate isomerase | F |
| Zwf | Glucose-6-phosphate dehydrogenase | F |
| *Proteases (GO:0051603 - Proteolysis involved in protein catabolic process) (9, 34)* | | |
| BepA | Metalloprotease | O |
| ClpA | ATP-dependent protease | O |
| ClpP | ATP-dependent protease | O |
| ClpX | ATP-dependent protease | O |
| DegP | Periplasmic serine endoprotease | O |
| HslU | ATP-dependent Protease | O |
| HslV | ATP-dependent Protease | O |
| Lon | ATP-dependent Protease | O |
| LoiP | Metalloprotease | O |
| *Persistence Driver Genes (34)* | | |
| DnaC | DNA replication protein | L |
| DnaT | Primosomal protein | L |
| Lon | ATP-dependent protease | O |
| PriA | Primosomal protein | L |
| RpoH | RNA polymerase 32 (sigma H) | K |
| SucA | 2-oxoglutarate dehydrogenase | F |
| SulA | Cell division inhibitor | D |
| YqgE | DUF179 domain-containing protein | K |
| *Persistence Marker Genes (34)* | | |
| CyaA | Adenylate cyclase | F |
| CysK | O-acetylserine sulfhydrylase | E |
| DadA | D-amino acid dehydrogenase | E |
| GroL | Chaperonin | O |
| IbpA | Small heat shock protein | O |
| IbpB | Small heat shock protein | O |
| IscS | Cysteine desulfurase | E |
| IscU | Iron-sulfur cluster assembly | C |
| LipA | Lipoyl synthase | H |
| MdtK | Multidrug efflux pump | P |
| MutM | DNA-formamidopyrimidine glycosylase | L |
| NarP | DNA-binding transcriptional dual regulator | K |
| OmpC | Outer membrane porin | M |
| PriA | Primosomal protein | L |
| RibB | 3,4-dihydroxy-2-butanone-4-phosphate synthase | H |
| Rmf | Ribosome modulation factor | J |
| SsrA | Transfer-messenger RNA | K |
| YhaM | Putative L-cysteine desulfidase | S |

* Listed Clusters of Orthologous Genes (COG): “Energy production and conversion” (C), “Cell cycle control, cell division, chromosome partitioning” (D), “Amino acid transport and metabolism” (E), “Nucleotide transport and metabolism” (F), “Carbohydrate transport and metabolism” (G), “Coenzyme transport and metabolism” (H), “Lipid transport and metabolism” (I), “Transcription” (K), “Replication, recombination and repair” (L), “Cell wall/membrane/envelope biogenesis” (M), “Post-translational modification, protein turnover, and chaperones” (O), “Function unknown” (S)
